# Improved effects of honokiol on temozolomide-induced autophagy and apoptosis of drug-sensitive and -tolerant glioma cells

**DOI:** 10.1186/s12885-018-4267-z

**Published:** 2018-04-03

**Authors:** Chung-Ching Chio, Kung-Yen Chen, Cheng-Kuei Chang, Jian-Ying Chuang, Chih-Chung Liu, Shing-Hwa Liu, Ruei-Ming Chen

**Affiliations:** 1 Department of Neurosurgery, Chi-Mei Medical Center, Tainan, Taiwan; 2 Graduate Institute of Medical Sciences, College of Medicine and Comprehensive Cancer Center, Taipei Medical University, 250 Wu-Hsing St., Taipei, 110 Taiwan; 3 Cellular Physiology and Molecular Image Research Center and Department of Anesthesiology, Wan-Fang Hospital, Taipei Medical University, Taipei, Taiwan; 4 Department of Neurosurgery, Shuang Ho Hospital, Taipei Medical University, Taipei, Taiwan; 5 Graduate Institute of Neural Regenerative Medicine, Taipei Medical University, Taipei, Taiwan; 6 Anesthesiology and Health Policy Research Center and Department of Anesthesiology, Taipei Medical University Hospital, Taipei Medical University, Taipei, Taiwan; 7 Institute of Toxicology, College of Medicine, National Taiwan University, Taipei, Taiwan

**Keywords:** Honokiol, Temozolomide, Glioma cells, Autophagy and apoptosis, Drug tolerance

## Abstract

**Background:**

Temozolomide (TMZ)-induced side effects and drug tolerance to human gliomas are still challenging issues now. Our previous studies showed that honokiol, a major bioactive constituent of *Magnolia officinalis* (Houpo), is safe for normal brain cells and can kill human glioma cells. This study was further aimed to evaluate the improved effects of honokiol and TMZ on drug-sensitive and -resistant glioma cells and the possible mechanisms.

**Methods:**

TMZ-sensitive human U87-MG and murine GL261 glioma cells and TMZ-resistant human U87-MR-R9 glioma cells were exposed to honokiol and TMZ, and cell viability and LC50 of honokiol were assayed. To determine the death mechanisms, caspase-3 activity, DNA fragmentation, apoptotic cells, necrotic cells, cell cycle, and autophagic cells. The glioma cells were pretreated with 3-methyladenine (3-MA) and chloroquine (CLQ), two inhibitors of autophagy, and then exposed to honokiol or TMZ.

**Results:**

Exposure of human U87-MG glioma cells to honokiol caused cell death and significantly enhanced TMZ-induced insults. As to the mechanism, combined treatment of human U87-MG cells with honokiol and TMZ induced greater caspase-3 activation, DNA fragmentation, cell apoptosis, and cell-cycle arrest at the G_1_ phase but did not affect cell necrosis. The improved effects of honokiol on TMZ-induced cell insults were further verified in mouse GL261 glioma cells. Moreover, exposure of drug-tolerant human U87-MG-R9 cells to honokiol induced autophagy and consequent apoptosis. Pretreatments with 3-MA and CLQ caused significant attenuations in honokiol- and TMZ-induced cell autophagy and apoptosis in human TMZ-sensitive and -tolerant glioma cells.

**Conclusions:**

Taken together, this study demonstrated the improved effects of honokiol with TMZ on autophagy and subsequent apoptosis of drug-sensitive and -tolerant glioma cells. Thus, honokiol has the potential to be a drug candidate for treating human gliomas.

## Background

Human gliomas, predominantly arising from the transformation of astrocytes, are the most common and aggressive brain tumors [1, 2]. According to the grading system of the World Health Organization, gliomas are classed into low-grade pilocytic astrocytomas and diffuse fibrillary astrocytomas with better prognoses (grades I and II) as well as high-grade anaplastic astrocytoma and glioblastoma multiforme (GBM) with worse prognoses (grades III and IV). Among these glioma types, GBMs are most lethal brain tumors [2, 3]. Because of uncontrolled tumor proliferation, infiltrative growth, angiogenesis, and resistance to apoptosis, the 5-year survival rate of malignant glioma patients is about 5% [4, 5]. In addition, the location of tumor occurrence in the brain and the high variability in genetic abnormalities are two other key risk factors existing in malignant gliomas [6]. The standard therapeutic approach for malignant gliomas is surgical resection followed by concurrent irradiation and adjuvant chemotherapy [1]. Unfortunately, the median overall survival rate of GBM is low at 10.2~ 14.6 months [4]. Therefore, malignant gliomas remain a challenge in the face of optimal treatment that includes surgery and chemotherapy.

Temozolomide (TMZ), a DNA-alkylating agent, is a chief chemotherapeutic drug for treating malignant gliomas [7]. With an excellent ability to penetrate the blood-brain barrier (BBB), TMZ can significantly induce apoptotic insults to glioma cells and suppress tumor growth. As to the mechanism, TMZ carries out its DNA-alkylating activity by specifically alkylating guanine at the O^6^ site, which results in mispairing during subsequent DNA replication, consequently inducing cell cycle arrest and ultimately apoptosis of glioma cells [7, 8]. At present, surgical resection followed by radiotherapy with daily oral TMZ is the standard therapeutic regimen for newly diagnosed malignant gliomas [9]. Unfortunately, TMZ administration can lead to multiple side effects, including nausea, vomiting, constipation, headaches, fatigue, loss of appetite, mouth sores, and hair loss [10]. These complications can reduce the therapeutic performance and quality of life of glioma patients. Furthermore, tolerance to TMZ by high-grade gliomas, especially in recurrent GBM patients, has recently become a serious issue. One of the main causes of TMZ resistance is greater expression and activation of O^6^-methylguanine-DNA methyltransferase (MGMT) [11]. Fan et al. reported that targeting MGMT has the potential for treating TMZ-tolerant gliomas [12]. As a result, exploring effective chemotherapeutic drugs for TMZ-resistant gliomas is critical to build up a de novo strategy for therapy of GBM patients.

Honokiol (2-(4-hydroxy-3-prop-2-enyl-phenyl)-4-prop-2-enyl-phenol), a small-molecule polyphenol, is a major bioactive constituent of the traditional Chinese medicine *Magnolia officinalis* (Houpo). Previous studies showed extensive application of honokiol for treating a variety of diseases such as anxiety and nervous disturbances, thrombotic stroke, typhoid fever, and dead muscles [13, 14]. Our previous study also showed penetration of honokiol across the BBB and its low toxicity to normal brain cells in vitro and in vivo [15]. Accordingly, we studied the effects of honokiol on inducing apoptotic insults to neuroblastoma cells and glioma cells via intrinsic mitochondria-dependent pathways [15, 16]. Recently, our findings further validated the benefits of honokiol on autophagic injury to neuroblastoma cells and glioma cells, and the molecular mechanisms occur via the p53/phosphatydilinositol-3-kinase (PI3K)/Akt/mammalian target of rapamycin (mTOR) signaling pathway [17, 18]. Moreover, Huang et al. reported that honokiol may inhibit sphere formation and xenograft growth of oral cancer stem cells [19]. Lai et al. discovered higher expression of MGMT in cancer stem-like side population cells sorted from GBM8401 glioma cells [20]. And also, co-treatment with honokiol and O^6^-benzylguanine, an MGMT inhibitor, may have killed those GBM cancer stem cells. Recently, we suggested that autophagic apoptosis induced by hypoxia may be applied as a new therapeutic strategy for treating glioma patients [21]. However, the combined effect of honokiol and TMZ for therapy of GBM patients is still not well known. Therefore, this study was designed to evaluate the improved effects of honokiol and TMZ on killing drug-sensitive and -resistant glioma cells and the possible mechanisms.

## Methods

### Cell culture and drug treatment

Human U87-MG glioma cells (catalog number: HTB-14), purchased from American Type Culture Collection (Manassas, VA, USA), and murine GL261 glioma cells, a kind gift from Dr. Rong-Tsun Wu (Institute of Biopharmaceutical Sciences, National Yang-Ming University, Taipei, Taiwan), were cultured in Dulbecco’s modified Eagle’s medium (DMEM; Gibco-BRL Life Technologies, Grand Island, NY, USA) supplemented with 10% fetal bovine serum (FBS), L-glutamine (2 mM), penicillin (100 IU/mL), streptomycin (100 mg/mL), sodium pyruvate (1 mM), and nonessential amino acids (1 mM) at 37 °C in a humidified atmosphere of 5% CO_2_. Glioma cells were grown to confluence before drug treatment. Honokiol acquired from Sigma (St. Louis, MO, USA), with a purity of > 98%, was freshly dissolved in dimethyl sulfoxide (DMSO). TMZ was obtained from Enzo Life Sciences (Farmingdale, NY, USA) and was dissolved in DMSO. Human U87-MG cells and murine GL261 cells were exposed to honokiol at different concentrations, TMZ at a clinically relevant concentration of 100 μM, and a combination of honokiol and TMZ for various time intervals. Control cells received DMSO only. Human and mouse glioma cells were pretreated with 3-methyladenine (3-MA, 1 mM) or chloroquine (CLQ, 20 μM), two inhibitors of cell autophagy, purchased from Sigma, before exposure to honokiol and TMZ as described previously [21].

### Preparation of human TMZ-resistant glioma cells

Human TMZ-resistant glioma cells were prepared and selected following a previously described method [22]. Human U87-MG glioma cells were cultured in DMEM containing 10% FBS, 100 μg/ml streptomycin sulfate, and 100 U/ml penicillin-G sodium at 37 °C and 5% CO_2_. Glioma cells (10^5^) were seeded into 12-well tissue culture plates and maintained in culture medium supplemented with TMZ at 50 μM. Two days after pretreatment with a low dose of TMZ, human U87-MG cells were subsequently dissociated with trypsin, seeded at limited dilutions (1/5~ 1-fold dilutions) in 96-well tissue culture plates, and then maintained in culture medium with 100 μM TMZ. When a surviving colony produced, cells were dissociated with trypsin and allowed to grow in the same well. Eleven TMZ-tolerant colonies were selected. Among these drug-resistant colonies, colony 9 (U87-MG-R9) was more malignant and had a more-rapid growth rate, so it was chosen for this study. Human U87-MG-R9 cells were treated with honokiol at different concentrations for various time intervals. And also, human U87-MG-R9 cells were pretreated with 1 mM 3-MA and 20 μM CLQ for 1 h and then exposed to honokiol for 72 h.

### Assays of cell morphology and cell viability

Cell viability was assayed using a colorimetric method as described previously [23]. Briefly, human U87-MG cells, human U87-MG-R9 cells, and murine GL261 cells were seeded in 96-well tissue culture plates at a density of 10^4^ cells/well overnight. After drug treatment, human glioma cells were cultured in new medium containing 3-(4,5-dimethylthiazol-2-yl)-2,5-diphenyltetrazoliumbromide (0.5 mg/ml) for a further 3 h. After reacting, the blue formazan products in human U87-MG cells, mouse GL261 cells, and human U87MG-R9 cells were dissolved in DMSO, and the optical densities were spectrophotometrically measured at a wavelength of 550 nm. Cell morphologies were observed and photographed using a light microscope (Nikon, Tokyo, Japan).

### Assay of caspase-3 activation

The enzyme activity of caspase-3 was assayed using a fluorometric substrate assay kit as described previously [24]. After drug administration, human U87-MG glioma cells were lysed using a buffer containing 1% Nonidet P-40, NaCl (200 mM), Tris/HCl (pH 7.4, 20 mM), and a cocktail of proteinase inhibitors, including leupeptin (10 mg/ml), aprotinin (0.27 U/ml), and phenylmethylsulfonyl fluoride (PMSF, 100 mm). Cell extracts (25 mg of total protein) were incubated with 50 mM of a specific fluorogenic peptide substrate in 200 ml of a cell-free system buffer composed of 10 mM 4-(2-hydroxyethyl)-1-piperazineethanesulfonic acid (HEPES, pH 7.4), 220 mM mannitol, 68 mM sucrose, 2 mM NaCl, 2.5 mM KH_2_PO_4_, 0.5 mM ethylene glycol tetraacetic acid (EGTA), 2 mM MgCl_2_, 5 mM pyruvate, 0.1 mM PMSF, and 1 mM dithiothreitol. The peptide substrate for the caspase-3 enzyme assay was DEVD. The peptide was conjugated to 7-amino-4-trifluoromethyl coumarin for fluorescence detection. Intensities of the fluorescent products were measured with a spectrometer.

### Quantification of DNA fragmentation

DNA fragmentation in human U87-MG glioma cells was quantified using a cellular DNA fragmentation enzyme-linked immunosorbent assay (ELISA) kit (Boehringer Mannheim, Indianapolis, IN, USA) as described previously [25]. Briefly, 2 × 10^5^ human U87-MG glioma cells were subcultured in 24-well tissue culture plates and labeled with 5-bromo-2’-deoxyuridine (BrdU) overnight. Cells were harvested and suspended in culture medium. One hundred microliters of the cell suspension was added to each well of 96-well tissue culture plates. Human U87-MG cells were cocultured with honokiol and TMZ for another 8 h at 37 °C in a humidified atmosphere of 5% CO_2_. Amounts of BrdU-labeled DNA in the cytoplasm were quantified using an Anthos 2010 microplate photometer (Anthos Labtec Instruments, Lagerhausstrasse, Wals/Salzburg, Austria) at a wavelength of 450 nm.

### Analysis of apoptotic cells

Apoptosis levels of human U87-MG cells, human U87-MG-R9 cells, and murine GL261 cells were determined using propidium iodide (PI) to detect DNA injury in nuclei according to a previously described method [26]. After drug administration, neuroblastoma cells were harvested and fixed in cold 80% ethanol. Following centrifugation and washing, fixed cells were stained with PI and analyzed using a FACScan flow cytometer (Becton Dickinson).

### Quantification of necrotic cells

Necrotic cells were quantified using a photometric immunoassay according to a previously described method [15]. Briefly, human U87-MG glioma cells (10^5^ cells) were seeded in 96-well tissue culture plates overnight. After drug administration, cell lysates and culture medium were collected, and necrotic cells were immunodetected using mouse monoclonal antibodies against histone. After an antibody reaction and washing, the colorimetric product was measured at 405 nm against a substrate solution as a blank.

### Assay of cell autophagy

Cell autophagy was analyzed by quantifying acidic vesicular organelles using flow cytometry as described previously [18]. After drug treatment, human U87-MG cells, human U87-MG-R9 cells, and murine glioma GL 261 cells (10^5^ cells) were treated with 1 μg/ml of acridine orange (AO) for 20 min. Then, cells were collected in phenol red-free DMEM. The green and red fluorescence levels of AO in cells were measured by flow cytometry (Becton Dickinson, San Jose, CA). Fluorescent intensities were quantified with the aid of CellQuest software (Becton Dickinson).

### Statistical analyses

The statistical significance of differences between groups was evaluated using a one-way analysis of variance (ANOVA) with Duncan’s multiple-range test. Differences were considered statistically significant at *p* values of < 0.05.

## Results

**Fig. 1:**
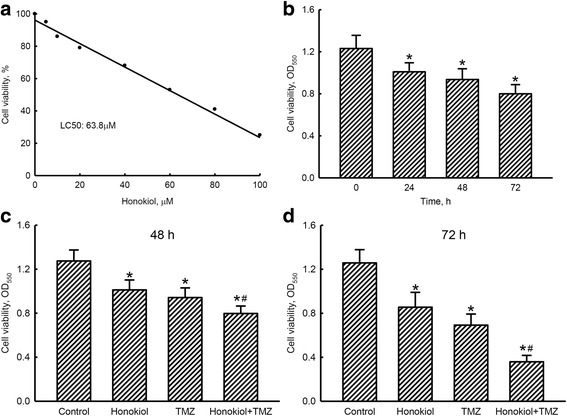
Effects of honokiol and temozolomide (TMZ) on the viability of human U87-MG glioma cells. After exposure to 1, 5, 10, 20, 40, 60, 80, and 100 μM honokiol for 72 h (**a**) and to 40 μM honokiol for 24, 48, and 72 h (**b**), the viability of human U87-MG cells was assayed using a colorimetric method. Human U87-MG cells were exposed to honokiol (40 μM), TMZ (100 μM), and their combination for 48 (**c**) and 72 h (**d**). Cell viability was assayed using a colorimetric method. Each value represents the mean ± SEM for *n* = 6. * and ^#^, values significantly (*p* < 0.05) differ from the respective control and TMZ-treated groups

After exposure to 5, 10, 20, 40, 60, 80, and 100 μM honokiol for 72 h, viability of human U87-MG glioma cells had respectively declined by 5, 14, 21, 32, 47, 59 and 75%, (Fig. 1a). The 50% lethal concentration (LC_50_) of honokiol to human U87-MG cells was 63.8 μM. Exposure of human glioma cells to 40 μM honokiol for 24, 48, and 72 h respectively caused significant 18, 34 and 35% decreases in cell viability (Fig. 1b). Treatment of human U87-MG cells with 40 μM honokiol or 100 μM TMZ respectively killed 21 and 26% of human glioma cells (Fig. 1c). In contrast, cotreatment with honokiol and TMZ for 48 h led to 37% of cells dying. After exposure to honokiol or TMZ for 72 h, the viability of human U87-MG cells respectively decreased by 32 and 45% (Fig. 1d). Cotreatment with honokiol and TMZ for 72 h significantly induced 71% cell death.

**Fig. 2:**
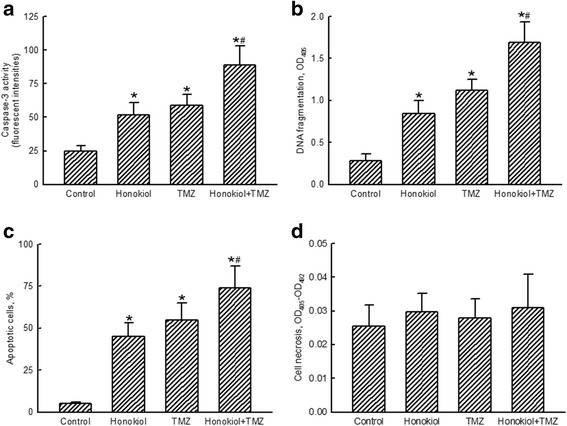
Improved effects of honokiol on temozolomide (TMZ)-induced caspase-3 activation, DNA fragmentation, cell apoptosis, and cell necrosis. Human U87-MG glioma cells were exposed to honokiol (40 μM), TMZ (100 μM), and their combination for 72 h. The activity of caspase-3 was assayed using a fluorometric substrate method (**a**). DNA fragmentation was quantified with a cellular DNA fragmentation ELISA kit (**b**). Apoptotic cells were quantified using flow cytometry (**c**). Necrotic cells were determined with a photometric immunoassay (**d**). Each value represents the mean ± SEM for *n* = 6. * and ^#^, values significantly (*p* < 0.05) differ from the respective control and TMZ-treated groups

Treatment of human glioma U87-MG cells with honokiol or TMZ for 72 h respectively augmented activities of caspase-3 by 2.1- and 2.4-fold (Fig. 2a). In comparison, cotreatment with honokiol and TMZ caused a 3.6-fold increase in activation of the caspase-3 enzyme. After exposure to honokiol or TMZ for 72 h, DNA fragmentation was respectively triggered by 3- and 4-fold (Fig. 2b). Cotreatment of human U87-MG cells with honokiol and TMZ led to 6-fold stimulation of DNA fragmentation (Fig. 2c). In parallel, honokiol or TMZ respectively induced 45 and 55% of human U87-MG cells to undergo apoptosis. Attractively, treatment with neither honokiol, TMZ, nor a combination of these two drugs influenced necrosis of human U87-MG glioma cells (Fig. 2d).

**Fig. 3:**
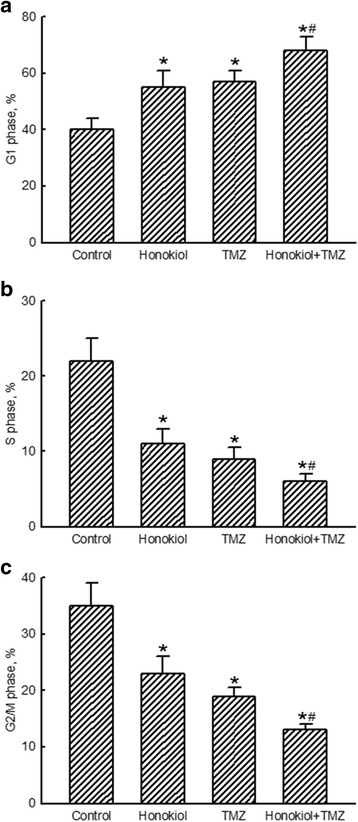
Enhanced effects of honokiol on temozolomide (TMZ)-induced cell cycle arrest. Human U87-MG glioma cells were exposed to honokiol (40 μM), TMZ (100 μM), and their combination for 72 h. Following centrifugation and washing, fixed cells were stained with propidium iodide and analyzed by flow cytometry. Proportions of human glioma cells at the G_1_ phase (**a**), S phase (**b**), and G_2_/M phase (**c**) were quantified. Each value represents the mean ± SEM for *n* = 6. * and ^#^, values significantly (*p* < 0.05) differ from the respective control and TMZ-treated groups

Exposure of human U87-MG cells to honokiol or TMZ respectively caused significant 38 and 43% enlargements in proportions of cells in the G_1_ phase (Fig. 3a). However, combined treatment with honokiol and TMZ enhanced proportions of human glioma cells arrested at the G_1_ phase by 70%. In contrast, portions of human U87-MG cells at the S phase were lessened by 50, 59 and 73% following exposure to honokiol, TMZ, and their combination, respectively (Fig. 3b). Also, treatment of human glioma cells with honokiol, TMZ, and their mixture respectively caused 34, 46 and 63% reductions in percentages of human U87-MG cells at the G_2_/M phase (Fig. 3c).

**Fig. 4:**
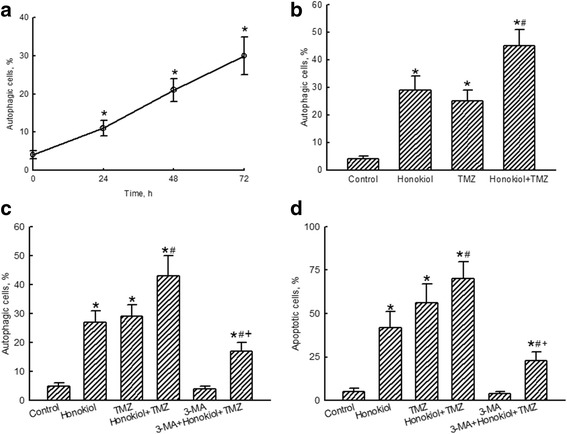
Effects of honokiol and temozolomide (TMZ) on autophagy of human U87-MG glioma cells. Human U87-MG cells were exposed to 40 μM honokiol for 24, 48, and 72 h (**a**) and to honokiol (40 μM), TMZ (100 μM), and their combination for 72 h (**b**). Cell autophagy was analyzed by flow cytometry. Cells were pretreated with 1 mM 3-methyladenine (3-MA), an inhibitor of cell autophagy, for 1 h and then exposed to honokiol (40 μM), TMZ (100 μM), and their combination for 72 h. Cell autophagy (**c**) and cell apoptosis (**d**) were quantified by flow cytometry. Each value represents the mean ± SEM for *n* = 6. *, ^#^, and ^+^, values significantly (*p* < 0.05) respectively differ from the control, TMZ-, and honokiol+TMZ-treated groups

Exposure of human glioma cells to 40 μM honokiol for 24, 48, and 72 h triggered cell autophagy by 11, 21%, and 30%, respectively (Fig. 4a). The proportions of human U87-MG cells suffering autophagic insults after administration of honokiol or TMZ respectively reached 29% and 25% (Fig. 4b). Nevertheless, combined treatment with honokiol and TMZ caused a noteworthy 45% stimulation in cell autophagy. Pretreatment of human U87-MG cells with 3-MA, an inhibitor of autophagy, slightly induced cell autophagy but led to a significant 66% attenuation in autophagic insults induced by the combination of honokiol and TMZ (Fig. 4c). In addition, the improved effect of honokiol and TMZ on triggering apoptosis of human glioma U87-MG cells was suppressed by 69% after pretreatment with 3-MA (Fig. 4d).

**Fig. 5:**
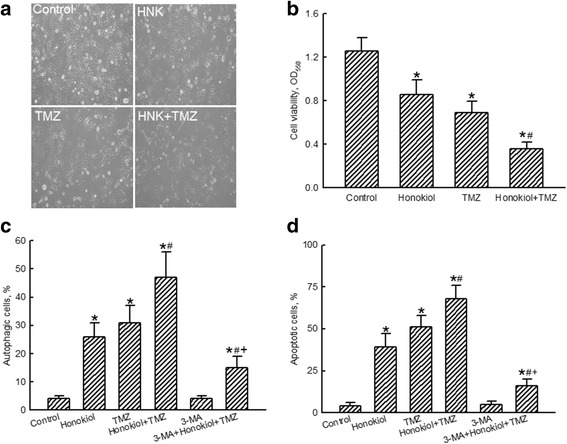
Effects of honokiol and temozolomide (TMZ) on insults to murine GL261 glioma cells. Murine GL261 cells were exposed to honokiol (40 μM), TMZ (100 μM), and their combination for 72 h. Cell morphology was observed by light microscopy (**a**). Cell viability was assayed using a colorimetric method (**b**). Cell autophagy was analyzed by flow cytometry. Murine GL261 glioma cells were pretreated with 1 mM 3-methyladenine (3-MA), an inhibitor of cell autophagy, for 1 h and then exposed to honokiol (40 μM), TMZ (100 μM), and their combination for 72 h. Cell autophagy (**c**) and cell apoptosis (**d**) were quantified using flow cytometry. Each value represents the mean ± SEM for *n* = 6. *, ^#^, and ^+^, values significantly (*p* < 0.05) respectively differ from the control, TMZ-, and honokiol+TMZ-treated groups

Exposure of GL261 cells to honokiol or TMZ individually for 72 h decreased the cell number and induced morphologic shrinkage (Fig. 5a). Combined treatment with honokiol and TMZ caused more-serious injury to GL261 cells. Treatment with honokiol or TMZ decreased viability of GL261 cells by 32 and 45%, respectively (Fig. 5b). In contrast, following treatment with a combination of honokiol and TMZ, viability of GL261 glioma cells declined by 71%. Exposure to honokiol, TMZ, and a mixture of these two drugs respectively led to significant 26, 31, and 47% inductions in autophagy of murine GL261 glioma cells (Fig. 5c). Pretreatment of GL261 cells with 3-MA alleviated cell autophagy in GL261 cells triggered by cotreatment with honokiol and TMZ by 68%. Apoptotic insults to GL261 glioma cells were respectively induced by 46, 56, and 70% after exposure to honokiol, TMZ, and their combination (Fig. 5d). Pretreatment with 3-MA led to a 67% lessening in apoptosis of murine glioma GL261 cells triggered by the mixture of honokiol and TMZ. Pretreatment of GL261 glioma cells with 3-MA did not affect cell autophagy or apoptosis (Fig. 5d).

**Fig. 6:**
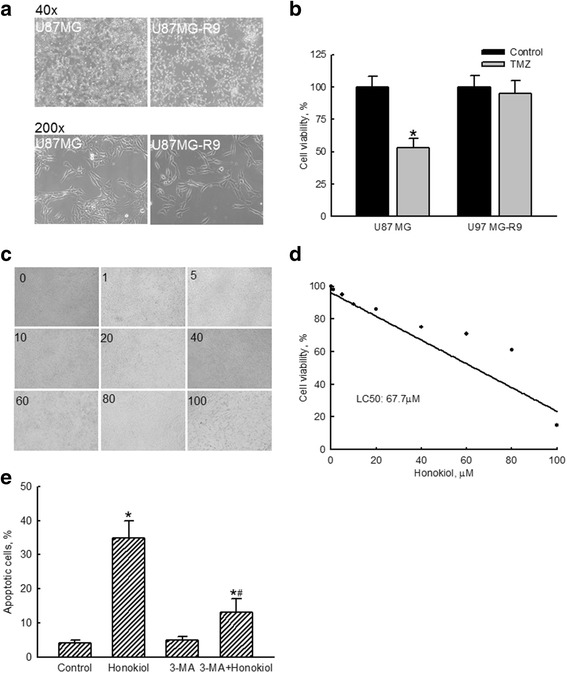
Effects of honokiol on killing temozolomide (TMZ)-resistant human glioma cells. Human TMZ-tolerant U87-MG-R9 cells were prepared by selection from human U87-MG glioma cells as described in "Materials and Methods". Morphologies of human U87-MG and U87-MG-R9 cells were observed and photographed (**a**). Cytotoxicity of TMZ at 100 μM to U87-MG and U87-MG-R9 cells was assayed using a colorimetric assay (**b**). Human U87-MG-R9 cells were exposed to 1, 5, 10, 20, 40, 60, 80, and 100 μM of honokiol for 72 h. Effects of honokiol on the cell number and morphology were evaluated (**c**). Cell viability was assayed using a colorimetric method (**d**). The 50% lethal concentration (LC_50_) was calculated. Human U87-MG-R9 cells were pretreated with 1 mM 3-methyladenine (3-MA), an inhibitor of cell autophagy, for 1 h and then exposed honokiol (40 μM) for 72 h. Cell apoptosis was quantified using flow cytometry (**e**). Each value represents the mean ± SEM for *n* = 6. *, the value significantly differs from the respective control, *p* < 0.05

Human U87-MG-R9 cells were prepared and selected from human U87-MG cells for further evaluation to see if honokiol could kill TMZ-resistant glioma cells (Fig. 6). Differential morphologies of human U87-MG glioma cells and human U87-MG-R9 TMZ-tolerant cells are shown in Fig. 6a. Exposure of human U87-MG cells to 100 μM TMZ for 72 h caused a 48% decrease in cell viability (Fig. 6b). In comparison, the viability of human U87-MG-R9 cells was not influenced following administration of 100 μM TMZ for 72 h. Treatment of human TMZ-resistant U87-MG-R9 cells with 1, 5, 10, 20, 40, 60, 80, and 100 μM honokiol for 72 h decreased cell numbers and changed cell morphologies (Fig. 6c). The LC_50_ of honokiol to human U87-MG-R9 cells was 67.7 μM (Fig. 6d). Exposure of U87-MG-R9 cells to 40 μM honokiol for 72 h induced cell apoptosis by 35% (Fig. 6e). Pretreatment of TMZ-resistant glioma cells with 3-MA did not trigger apoptotic insults but caused a significant 63% reduction in honokiol-induced cell apoptosis.

**Fig. 7:**
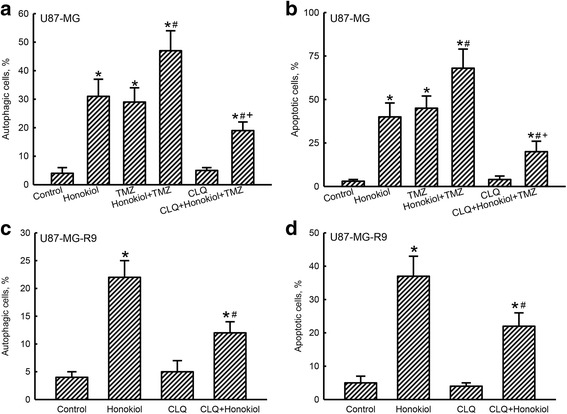
Effects of chloroquine (CLQ), an inhibitor of cell autophagy, on honokiol-induced autophagy and apoptosis in human temozolomide (TMZ)-sensitive and -resistant glioma cells. Human U87-MG cells were pretreated with 20 μM CLQ for 1 h and then exposed to 40 μM honokiol, 100 μM TMZ, and their combination for another 72 h (**a**, **b**). Human TMZ-resistant U87-MG-R9 cells, selecting from human U87-MG cells and culturing in DMEM medium containing TMZ, were pretreated with 20 μM CLQ for 1 h and then exposed to 40 μM honokiol for 72 h (**c**, **d**). Cell autophagy (**a** and **c**) and apoptosis (**b** and **d**) were quantified using flow cytometry. Each value represents the mean ± SEM for *n* = 3. Symbols *, ^#^, and ^+^, indicate the values significantly (*p* < 0.05) differ from the control, TMZ-, and honokiol+TMZ-treated groups, respectively

CLQ was further applied into human TMZ-sensitive and resistant glioma cells to confirm honokiol-induced cell autophagy and apoptosis (Fig. 7). Exposure of human U87-MG cells to honokiol induced cell autophagy and apoptosis by 31 and 40%, respectively (Fig. 7a, b). Treatment with TMZ led to 29 and 45% of U87-MG cells undergoing to autophagy and apoptosis. The percentages of human U87-MG cells undergoing to autophagy and apoptosis were respectively elevated to 47 and 68% following a combined treatment of honokiol and TMZ (Fig. 7a, b). In contrast, pretreatment with CLQ did not induce cell insults but significantly lowered honokiol+TMZ-induced cell autophagy and apoptosis by 60 and 71%, respectively (Fig. 7a, b). In human TMZ-resistant U87-MG-R9 cells, honokiol induced cell autophagy and apoptosis by 22 and 37% (Fig. 7c, d). However, pretreatment with CLQ did not cause injury to human TMZ-tolerant glioma cells, but descended honokiol-induced cell autophagy and apoptosis by 45 and 41%, respectively (Fig. 7c, d).

## Discussion

This study shows the improved effects of honokiol on TMZ-induced death of drug-sensitive and -resistant malignant glioma cells. Gliomas are major solid tumors that occur in the brain, accounting for about 80% of all malignant brain tumors [1]. Our present results revealed the benefits of honokiol at 40 μM, which was less than its LC_50_ of 63 μM, to efficiently kill human and murine malignant glioma cells. Therapeutic options for treating brain diseases are limited because of the presence of the blood-brain barrier (BBB). The presence of the BBB is an important limitation when exploring new therapeutic options for treating brain diseases [27]. Our previous findings also validated that honokiol can pass through the BBB in vitro and in vivo [15]. Furthermore, the safety of honokiol to normal brain cells was proven, including human HA-h astrocytes and mouse cerebrovascular endothelial cells. Thus, honokiol is a good candidate drug for treating malignant gliomas. TMZ is a first-line chemotherapeutic drug for GBM patients [9]. However, TMZ administration has multiple side effects such as nausea, vomiting, constipation, headaches, fatigue, loss of appetite, mouth sores, and hair loss [10]. Human malignant gliomas are very aggressive, and the 5-year survival rate of malignant glioma patients is about 5% [4]. Fascinatingly, our present results designated the enhanced effects of honokiol on TMZ-induced death of human and murine glioma cells. As a result, honokiol has specific benefits of reducing complications of TMZ by decreasing its dosage and accordingly improving the life quality of GBM patients.

Honokiol-involved improved effects on TMZ-induced insults to malignant glioma cells occur via cell cycle arrest at sub-G_1_ phase and cell apoptosis. In parallel with lessening the viability of human U87-MG glioma cells, treatment with honokiol or TMZ individually led to cell shrinkage, caspase-3 activation, DNA fragmentation, and cell cycle arrest at the sub-G_1_ phase. Moreover, combined treatment with honokiol and TMZ induced greater cell insults. Such improved actions induced by honokiol were also confirmed in murine GL261 glioma cells. Characteristically, a shrunken morphology, caspase-3 activation, DNA fragmentation, and cell cycle arrest at the sub-G_1_ phase are significant features of cells undergoing apoptosis [28, 29]. Hence, honokiol significantly kills glioma cells with TMZ via an apoptotic pathway. In addition, the present results revealed augmentation in the proportions of human U87-MG cells arrested at the G_1_ phase. Gelbert et al. reported that treatment of colorectal cancer and acute myeloid leukemia cells with LY2835219, a CDK4/6 inhibitor, resulted in G_1_ phase arrest, cell apoptosis, and further suppression of tumor growth in tumor xenografts [30]. Our previous study also demonstrated the effects of honokiol on inducing glioma cell apoptosis via p53/p21-mediated cell cycle arrest at the G_1_ phase [16]. Therefore, combined treatment with honokiol and TMZ can induce apoptotic insults to malignant glioma cells through a cell cycle-arrest mechanism.

Co-treatment with honokiol and TMZ led to autophagy and subsequent apoptosis in human and mouse malignant glioma cells. Honokiol increased the proportions of human U87-MG cells with acidic vesicular organelles. Acidic vesicular organelles are one of the consistent characteristics indicating cell autophagy [31]. In addition, our previous study has shown that honokiol could increase levels of microtubule-associated protein light chain 3 (LC3)-II, a consistent marker of autophagy, in human U87-MG cells and glioma tissues of nude mice via activation of the p53/PI3K/Akt/mTOR signaling pathway (18). Moreover, the honokiol-induced autophagy was attenuated by 3-MA and CLQ, two typical inhibitors of autophagy. Thus, honokiol can trigger autophagy of human malignant glioma cells. TMZ causes cell apoptosis by specifically triggering alkylation of guanine at the O^6^ site [7, 8]. Recently, Shen et al. recently showed that TMZ can induce cell autophagy, thereby suppressing the development of glioblastomas [32]. Herein, we proved TMZ-induced autophagic insults to human and murine glioma cells. Honokiol may boost the TMZ-induced autophagy of malignant glioma cells. Remarkably, the honokiol-induced improvement in apoptosis of human and mouse glioma cells simultaneously decreased after suppressing cell autophagy with 3-MA or CLQ. Our previous study proposed a de novo strategy for treating gliomas via hypoxia-induced autophagic apoptosis [21]. In addition, numerous of autophagy-related proteins participate in mediation of mitochondrial fusion [33]. Pillai et al. showed that honokiol could induce mitochondrial fusion by activating mitochondrial Sirt3 in cardiomyocytes [34]. Mitochondrial fusion is associated with a respiratory phenotype. Disturbance of mitochondrial electron transport chain function could induce autophagy and apoptosis [35]. Another reason explaining honokiol-induced cell autophagy and apoptosis may be via triggering expression of autophagic proteins in function mitochondria. Our present study implied the potential of honokiol for treating glioma patients with TMZ by significantly inducing cell autophagy and subsequent apoptosis.

Honokiol induces autophagic apoptosis of human TMZ-resistant glioma cells. Despite TMZ being a first-line chemotherapeutic drug in standard concurrent chemoradiotherapy for glioma patients, drug resistance is still a common and serious issue in the clinic [9, 11]. In this study, we successfully prepared TMZ-resistant human glioma cells. Compared to drug-sensitive U87-MG cells, U87-MG-R9 cells were tolerant of TMZ treatment. In contrast, administration of honokiol to TMZ-resistant glioma cells decreased cell viability in a time-dependent manner. Gerson proffered one of the main reasons to explain TMZ tolerance of MGMT induction and activation [11]. Fan et al. further stated that targeting MGMT may be beneficial for treating TMZ-tolerant gliomas [12]. Recently, Lai et al. found that GBM cancer stem cells possess higher expression of MGMT so they are resistant to TMZ administration [20]. Cotreatment with honokiol and O^6^-benzylguanine, an MGMT inhibitor, caused effective cytotoxicity to those GBM cancer stem cells. However, this study showed that honokiol alone can kill TMZ-tolerant glioma cells. As to the mechanisms, honokiol induced apoptosis of human U87-MG-R cells. Pretreatments with 3-MA and CLQ decreased honokiol-induced cell autophagy and apoptosis. Thus, the mechanisms explaining honokiol-induced insults to human TMZ-tolerant glioma cells may be related to induction of autophagy and subsequent apoptosis. This is the first study to prove the effects of honokiol in killing TMZ-resistant glioma cells.

## Conclusions

In summary, this study determined that the LC_50_ of honokiol to human U87-MG glioma cells was 63.8 μM. Exposure of human glioma cells to 40 μM honokiol decreased cell viability in a time-dependent manner. Interestingly, honokiol induced an improved effect on TMZ-induced death of human malignant glioma cells. As to the mechanisms, combined treatment with honokiol and TMZ induced greater caspase-3 activation, DNA fragmentation, cell cycle arrest at the G_1_ phase, and cell apoptosis but did not influence cell necrosis. In addition, honokiol significantly triggered TMZ-induced cell autophagy in human glioma U87-MG cells. Pretreatment with 3-MA and CLQ caused significant alleviations of cell autophagy and consequent apoptosis induced by the combination of honokiol and TMZ. The improved effects of honokiol on TMZ-induced autophagy, apoptosis, and cell death were further confirmed using mouse glioma GL261 cells as another experimental model. Treatment of TMZ-resistant human glioma cells with honokiol decreased cell viability and induced cell apoptosis. 3-MA and CLQ could reduce the honokiol-induced autophagic and apoptotic insults to TMZ-tolerant human glioma cells. Taken together, this study demonstrated that honokiol can kill TMZ-sensitive and -resistant glioma cells through autophagy and subsequent apoptosis. Thus, honokiol has the potential to be a candidate drug for therapy of human malignant gliomas. However, as this is an in vitro study, additional animal and translational investigations will be further designed and performed in the future.

## Abbreviations

3-MA: 3-methyladenine
AO: Acridine orange
BBB: Blood-brain barrier
BrdU: 5-bromo-2′-deoxyuridine
CLQ: Chloroquine
DMEM: Dulbecco’s modified Eagle’s medium
DMSO: Dimethyl sulfoxide
ELISA: Fragmentation enzyme-linked immunosorbent assay
FBS: Fetal bovine serum
GBM: Glioblastoma multiforme
LC50: Lethal concentration 50%
MGMT: O^6^-methylguanine-DNA methyltransferase
mTOR: Mammalian target of rapamycin
PI: Propidium iodide
PMSF: Phenylmethylsulfonyl fluoride
TMZ: Temozolomide

## References

[1] Brown TJ, Brennan MC, Li M, Church EW, Brandmeir NJ, Rakszawski KL, Patel AS, Rizk EB, Suki D (2016). Association of the extent of resection with survival in glioblastoma: a systematic review and meta-analysis. JAMA Oncol.

[2] Huse JT, Wallace M, Aldape KD, Berger MS, Bettegowda C, Brat DJ, Cahill DP, Cloughesy T, Haas-Kogan DA (2014). Where are we now? And where are we going? A report from the accelerate brain cancer cure (ABC2) low-grade glioma research workshop. Neuro-Oncol.

[3] Jordan JT, Gerstner ER, Batchelor TT, Cahill DP, Plotkin SR (2016). Glioblastoma care in the elderly. Cancer.

[4] Kageji T, Nagahiro S, Mizobuchi Y, Matsuzaki K, Nakagawa Y, Kumada H (2014). Boron neutron capture therapy (BNCT) for newly-diagnosed glioblastoma: comparison of clinical results obtained with BNCT and conventional treatment. J Med Investig.

[5] Xie Q, Mittal S, Berens ME (2014). Targeting adaptive glioblastoma: an overview of proliferation and invasion. Neuro-Oncol.

[6] Curtin JF, Liu N, Candolfi M, Xiong W, Assi H, Yagiz K, Edwards MR, Michelsen KS, Kroeger KM (2009). HMGB1 mediates endogenous TLR2 activation and brain tumor regression. PLoS Med.

[7] Friedman HS, Kerby T, Calvert H (2000). Temozolomide and Treatment of malignant glioma. Clin Cancer Res.

[8] Alonso MM, Gomez-Manzano C, Bekele BN, Yung WK, Fueyo J (2007). Adenovirus-based strategies overcome temozolomide resistance by silencing the O6-methylguanine-DNA methyltransferase promoter. Cancer Res.

[9] Stupp R, Mason WP, van Den Bent MJ, Weller M, Fisher B, Taphoorn MJ, Belanger K, Brandes AA, Marosi C (2005). Radiotherapy plus concomitant and adjuvant temozolomide for glioblastoma. N Engl J Med.

[10] Omuro A, DeAngelis LM (2013). Glioblastoma and other malignant gliomas: a clinical review. JAMA.

[11] Gerson SL (2002). Clinical relevance of MGMT in the treatment of cancer. J Clin Oncol.

[12] Fan CH, Liu WL, Cao H, Wen C, Chen L, Jiang G (2013). O^6^-methylguanine DNA methyltransferase as a promising target for the treatment of temozolomide-resistant gliomas. Cell Death Dis.

[13] Chen L, Zhang Q, Yang G, Fan L, Tang J, Garrard I, Ignatova S, Fisher D, Sutherland IA (2007). Rapid purification and scale-up of honokiol and magnolol using high-capacity high-speed counter-current chromatography. J Chromatogr A.

[14] Fried LE, Arbiser JL (2009). Honokiol, a multifunctional antiangiogenic and antitumor agent. Antioxid Redox Signal.

[15] Lin JW, Chen JT, Hong CY, Lin YL, Wang KT, Yao CJ, Lai GM, Chen RM (2012). Honokiol traverses the blood-brain barrier and induces apoptosis of neuroblastoma cells via an intrinsic Bax-mitochondrion-cytochrome c-caspase protease pathway. Neuro-Oncol.

[16] Lin CJ, Chang YA, Lin YL, Chio CC, Chen RM (2016). Preclinical effects of honokiol on treating glioblastoma multiforme via G1 phase arrest and cell apoptosis. Phytomedicine.

[17] Yeh PS, Wang W, Chang YA, Lin CJ, Wang JJ, Chen RM (2016). Honokiol induces autophagy of neuroblastoma cells through activating the PI3K/Akt/mTOR and endoplasmic reticular stress/ERK1/2 signaling pathways and suppressing cell migration. Cancer Lett.

[18] Lin CJ, Chen TL, Tseng YY, Wu GJ, Hsieh MH, Lin JW, Chen RM (2016). Honokiol induces autophagic cell death in malignant glioma through reactive oxygen species-mediated regulation of the p53/PI3K/Akt/mTOR signaling pathway. Toxicol Appl Pharmacol.

[19] Huang JS, Yao CJ, Chuang SE, Yeh CT, Lee LM, Chen RM, Chao WJ, Whang-Peng J, Lai GM (2016). Honokiol inhibits sphere formation and xenograft growth of oral cancer side population cells accompanied with JAK/ STAT signaling pathway suppression and apoptosis induction. BMC Cancer.

[20] Lai IC, Shih PH, Yao CJ, Yeh CT, Wang-Peng J, Lui TN, Chuang SE, Hu TS, Lai TY, Lai GM (2015). Elimination of cancer stem-like cells and potentiation of temozolomide sensitivity by Honokiol in glioblastoma multiforme cells. PLoS One.

[21] Cheng BC, Chen JT, Yang ST, Chio CC, Liu SH, Chen RM (2017). Cobalt chloride treatment induces autophagic apoptosis in human glioma cells via a p53-dependent pathway. Int J Oncol.

[22] Stepanenko AA, Andreieva SV, Korets KV, Mykytenko DO, Baklaushev VP, Huleyuk NL, Kovalova OA, Kotsarenko KV, Chekhonin VP (2016). Temozolomide promotes genomic and phenotypic changes in glioblastoma cells. Cancer Cell Int.

[23] Lin CJ, Lin YL, Luh F, Yen Y, Chen RM (2016). Preclinical effects of CRLX101, an investigational camptothecin-containing nanoparticle drug conjugate, on treating glioblastoma multiforme via apoptosis and antiangiogenesis. Oncotarget.

[24] Wu GJ, Wang W, Lin YL, Liu SH, Chen RM (2016). Oxidative stress-induced apoptotic insults to rat osteoblasts are attenuated by nitric oxide pretreatment via GATA-5-involved regulation of Bcl-X_L_ gene expression and protein translocation. Arch Toxicol.

[25] Wu GJ, Chen JT, Tsai HC, Chen TL, Liu SH, Chen RM. Protection of dexmedetomidine against ischemia/reperfusion-induced apoptotic insults to neuronal cells occurs via an intrinsic mitochondria-dependent pathway. J Cell Biochem. 2017;118(9):2635–44. 10.1002/jcb.25847 27987330

[26] Chang CY, Lui TN, Lin JW, Lin YL, Hsing CH, Wang JJ, Chen RM (2016). Roles of microRNA-1 in hypoxia-induced apoptotic insults to neural cells. Arch Toxicol.

[27] Doolittle ND, Anderson CP, Bleyer WA, Cairncross JG, Cloughesy T, Eck SL, Guastadisegni P, Hall WA, Muldoon LL (2001). Importance of dose intensity in neuro-oncology clinical trials: summary report of the sixth annual meeting of the blood-brain barrier disruption consortium. Neuro-Oncologia.

[28] Brandsma D, Stalpers L, Taal W, Sminia P, van den Bent MJ (2008). Clinical features, mechanisms, and management of pseudoprogression in malignant gliomas. Lancet Oncol.

[29] Chuang CY, Chen TL, Cherng YG, Tai YT, Chen TG, Chen RM (2011). Lipopolysaccharide induces apoptotic insults to human alveolar epithelial A549 cells through reactive oxygen species-mediated activation of an intrinsic mitochondrion-dependent pathway. Arch Toxicol.

[30] Gelbert LM, Cai S, Lin X, Sanchez-Martinez C, del Prado M, Lallena MJ, Torres R, Ajamie RT, Wishart GN (2014). Preclinical characterization of the CDK4/6 inhibitor LY2835219: in-vivo cell cycle-depen- dent/independent anti-tumor activities alone/in combination with gemcitabine. Investig New Drugs.

[31] Klionsky DJ, Abdelmohsen K, Abe A, Abedin MJ, Abeliovich H, Acevedo Arozena A, Adachi H, Adams CM, Adams PD (2012). Guidelines for the use and interpretation of assays for monitoring autophagy. Autophagy.

[32] Shen X, Kan S, Hu J, Li M, Lu G, Zhang M, Zhang S, Hou Y, Chen Y, Bai Y (2016). EMC6/TMEM93 suppresses glioblastoma proliferation by modulating autophagy. Cell Death Dis.

[33] Arbiser JL, Bonner MY, Gilbert LC (2017). Targeting the duality of cancer. NPJ Precis Oncol.

[34] Trotta AP, Gelles JD, Serasinghe MN, Loi P, Arbiser JL, Chipuk JE (2017). Disruption of mitochondrial electron transport chain function potentiates the pro-apoptotic effects of MAPK inhibition. J Biol Chem.

[35] Pillai VB, Samant S, Sundaresan NR, Raghuraman H, Kim G, Bonner MY, Arbiser JL, Walker DI, Jones DP, Gius D, Gupta MP (2015). Honokiol blocks and reverses cardiac hypertrophy in mice by activating mitochondrial Sirt3. Nat Commun.

